# Weed Growth Stage Estimator Using Deep Convolutional Neural Networks

**DOI:** 10.3390/s18051580

**Published:** 2018-05-16

**Authors:** Nima Teimouri, Mads Dyrmann, Per Rydahl Nielsen, Solvejg Kopp Mathiassen, Gayle J. Somerville, Rasmus Nyholm Jørgensen

**Affiliations:** 1 Department of Engineering-Signal Processing, Faculty of Science and Technology, Aarhus University, DK-8000 Aarhus C, Denmark; n.teimouri@eng.au.dk (N.T.); rnj@eng.au.dk (R.N.J.); 2 Department of Biosystems Engineering, University of Tehran, Tehran 1417466191, Iran; 3 IPM Consult ApS, DK-4295 Stenlille, Denmark; per.rydahl@ipmconsult.dk; 4 Department of Agroecology, Aarhus University, DK-4200 Slagelse, Denmark; sma@agro.au.dk (S.K.M.); gs@agro.au.dk (G.J.S.)

**Keywords:** computer vision, growth stage, leaf counting, convolutional neural network, deep learning

## Abstract

This study outlines a new method of automatically estimating weed species and growth stages (from cotyledon until eight leaves are visible) of in situ images covering 18 weed species or families. Images of weeds growing within a variety of crops were gathered across variable environmental conditions with regards to soil types, resolution and light settings. Then, 9649 of these images were used for training the computer, which automatically divided the weeds into nine growth classes. The performance of this proposed convolutional neural network approach was evaluated on a further set of 2516 images, which also varied in term of crop, soil type, image resolution and light conditions. The overall performance of this approach achieved a maximum accuracy of 78% for identifying *Polygonum* spp. and a minimum accuracy of 46% for blackgrass. In addition, it achieved an average 70% accuracy rate in estimating the number of leaves and 96% accuracy when accepting a deviation of two leaves. These results show that this new method of using deep convolutional neural networks has a relatively high ability to estimate early growth stages across a wide variety of weed species.

## 1. Introduction

To reduce the use of herbicides in agriculture, farmers must have knowledge of the condition of weeds in their field, in order to spray optimally, whilst minimizing the herbicide consumption. A compilation of the current top five factors for selecting herbicides and dosages lists “determination of weed species” and “determination of classes of weed growth stage” as Nos. 2 and 3 in importance [1]. Across major crops in Denmark, there is an unexploited potential to achieve a 20–40% reduction in herbicide use, whilst maintaining weed control, by targeting specific weeds, in situ [1]. However, a sociological study has shown that Danish farmers are reluctant to conduct field scouting and that recognition of weeds (in various growth stages) is a major obstacle to targeted weed control [2]. Morphological plant traits such as size, number of leaves and leaf shape are affected by many factors, including genes and environmental factors (soil texture, soil humidity, nutrient availability, temperature, light, humidity). Additionally, attack by fungi and pests can alter the size, shape and growth of plants.

Over time, several standards have been used to divide plants into discrete growth stages, based on the number of leaves and tillers. In early growth stages, the number of leaves is directly related to the growth stage; therefore, it is possible to determine the number of leaves and use this to identify the growth stage of young weeds. This knowledge of growth stage can be combined with knowledge of herbicide rates, meaning that more effective and efficient weed control can be implemented [3]. However, counting the leaves of weeds by using non-destructive and automatic methods such as computer vision is a challenge with which researchers are still grappling [4].

One of the main topics in an automatic system of precision weed control is the combination of machine vision and machine learning. An automated system should be able to identify the various weed species and detect the number of leaves with acceptable accuracy [5].

In order to make a robust system for identifying weed species and counting leaves, images need to cover the natural variation in terms of environmental conditions and plant development stages. These conditions include light settings, soil types and plant stress. The ideal system should be able to detect precisely the number of leaves of weed plants prior to applying weed management. An important problem in automatic leaf counting is that weed leaves frequently overlap each other and may be partly covered by the crop, yet all leaves must be counted in order to determine the current growth stage and select the correct treatment. However, manual identification of the number of leaves in weed images can be difficult, even when undertaken by experts [6].

Most automatic systems for counting leaves using computer vision are limited to binary images, meaning that the images must first be segmented from the background, and only once this is achieved can the number of leaves be counted [7,8,9,10]. A problem with methods relying on segmented plants is their inability to successfully process images where plants overlap each other. Giuffrida et al. [9] proposed a method for counting the number of leaves whereby the images were transferred from an RGB space to a log-polar space. The related properties of these new log-polar images were extracted, and a vector regression method was applied to count the number of leaves. One of the limitations of using a log-polar space is the necessity of using segmented images in both the training phase and in the later evaluation of the final model; this means that the use of log-polar extraction would be difficult to implement as an automatic system.

In recent years, convolutional neural networks (CNNs) have shown considerable success in computer vision and machine learning areas [11], because of their ability to extract efficient features for classifying images. CNNs have been widely applied for solving problems in the agricultural domain including plant species classification [12,13], weed detection [14,15], pest image classification [16] and plant disease detection and diagnosis [17]. Ren and Zemel [18] and Romera-Paredes and Torr [19] demonstrated how recursive neural networks can be used, for example, as a tool for segmentation in various domains including leaf-segmentation on the CVPPP LSC dataset [20], where the methods achieved promising results. However, these methods require training images, where leaves are fully segmented on an instance level, which can take several minutes to do precisely for each plant. Aich and Stavness [21] used the same dataset to demonstrate an encoder-decoder convolutional neural network, which used regression to count the number of leaves.

In contrast, the aim of this research is to develop an effective method for counting the number of leaves on plants from images taken in fields (including the cotyledon leaves) across 18 different species or families of weeds. The method presented in this study is based on a convolutional neural network that was trained to map images of different weeds across nine different growth-classes.

## 2. Data Material

**Figure 1:**
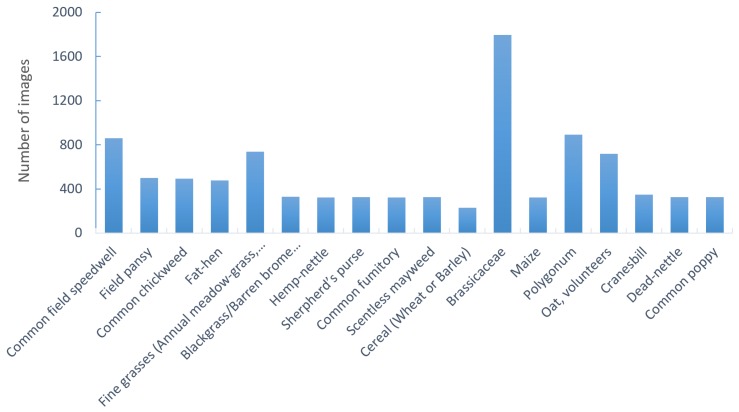
Different weed species and the number of samples in the training procedure.

**Figure 2:**
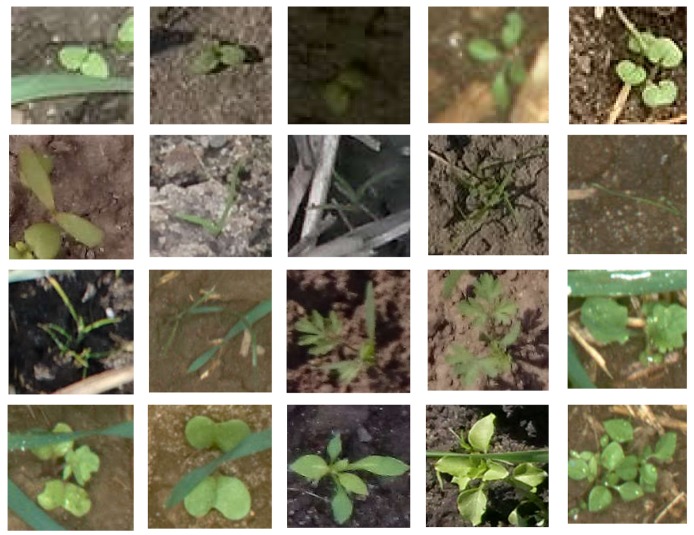

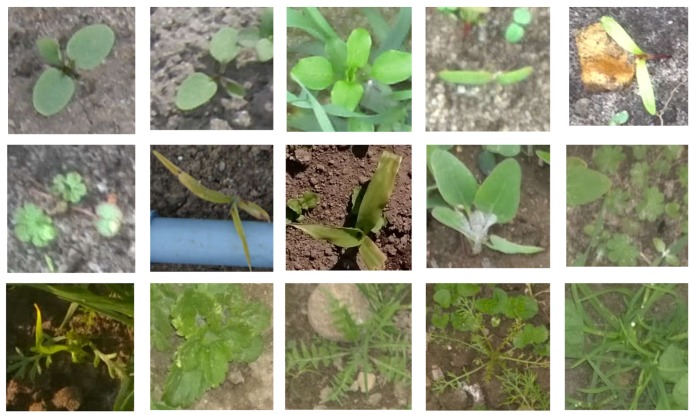
A random selection from the image datasets.

**Figure 3:**
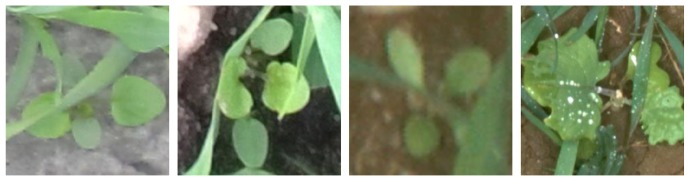
Samples of difficult images, where not all leaves are fully visible due to overlapping leaves.

As well as an acceptable method to identify leaf number when plants overlap each other, one of the objectives of this research was to loosen the requirements for the quality of cameras, meaning that it will no longer be necessary to use industrial cameras (such as NIR cameras) for counting the number of leaves on weeds. In this study, we looked at using RGB images from Nokia and Samsung cell phone cameras, from Samsung, Nikon, Canon and Sony consumer cameras and from a Point Grey industrial camera. These images were collected during three growing seasons and cover a total of 18 weed species or families (Figure 1). These images were taken in various cropping fields across all regions of Denmark; thereby covering a range of soil types, image resolutions and light conditions (Figure 2). One important factor that may influence the ability of the convolutional neural network to count the number of leaves is plants overlapping each other. Cases of partly-hidden weeds are shown in Figure 3. Images of overlapping individual plants can automatically be extracted from images using weed detection algorithms such as the fully-convolutional weed detector proposed by Dyrmann [14]. In order to make a convolutional network robust when examining images with occluded leaves, it must be presented with images containing overlapping leaves during the training phase. A total of 9649 image samples was acquired for the various weed species, with each image manually classified in terms of species and growth stage by experts. Two standards exist for counting leaves: one where only the true leaves are counted, and one where both cotyledon leaves and true leaves are counted. Here, the number of leaves includes the cotyledon leaves. The nine classes used here are 1-leaf, 2-leaves, 3-leaves, 4-leaves, 5-leaves, 6-leaves, 7-leaves, 8-leaves and >8-leaves. The images are publicly available at https://vision.eng.au.dk/leaf-counting-dataset/.

## 3. Methods

Traditional neural networks are composed of an input layer, several hidden layers with a limited number of neurons and an output layer. Because of these simple structure, they often require a manually-designed feature extraction, prior to the input layer. By contrast, deep convolutional neural networks (CNNs) are mainly distinguished from standard neural networks by their depth and weight sharing (between nodes in a layer); in CNNs, each layer’s weights are trained on the features from the previous layer’s output [22]. This ability to extract thousands of features automatically means that convolutional neural networks are able to classify images collected in uncontrolled conditions with a significantly lower error rate than previous classifier methods [23]. The layers within a CNN can consist of a number of convolutional and subsampling filters (used for automatic feature extraction), optionally followed by fully-connected layers, as in a standard multilayer neural network. When training on RGB image data, the input for the CNN will normally be a batch of images, which can be preprocessed in order to increase the variation.

### 3.1. Image Preprocessing

In this research, a CNN was used for counting the number of leaves on 18 different weed species or families. However, deep CNNs need to be trained on a large number of images in order to learn to extract general features automatically from the input data. Furthermore, a large number of images helps to regulate the network and reduce the risk of overfitting [22]. Overfitting happens when the network weights fit too well on the training set, and the network cannot then detect significant discriminative features within new images. Overfitting makes it difficult for the network to generalize to new examples that were not in the training set. There are various strategies to prevent overfitting, including increasing the number of training images or adding ‘dropout’ (explained in Section 3.2.4). In this study, the training dataset was increased (without the need for extra manual annotation) by using horizontal-flip, rotation, zoom, width shift, height shift and Gaussian smoothing filters. This data augmentation created a training dataset containing 11,907 images within the training dataset.

### 3.2. Network Architecture

**Figure 4:**
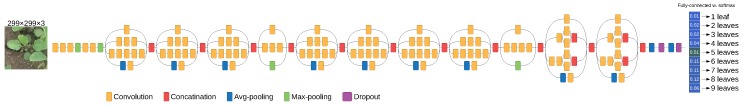
Inception-v3 architecture with modified last layers.

There are various pre-trained network architectures that can be used for image classification, but they have often been trained with a huge number of images (such as ImageNet [24]), which are very different from plant images. However, even though plant images are very different from the images in the ImageNet dataset, general features learned from ImageNet data can be adapted to plant images with relatively few training iterations. By fine-tuning the existing network weights, rather than starting with totally random weights, the network was stimulated to learn general features, rather than overfitting. Common pre-trained networks include AlexNet [25], GoogLeNet [26], ResNet [27] and VGG [28]. We selected the Inception-v3 (Figure 4) architecture, which is a refinement of the GoogLeNet architecture. The Inception-v3 architecture was selected due to its good performance, ease of implementation and relatively low computational cost, which enabled it to obtain excellent results in the 2015 ImageNet competition [29].

The Inception-v3 architecture contains so-called inception modules, which combine pooling layers with filters of various sizes, allowing them to utilize the benefit of each filter size: wide filters (5 × 5) are able to extract context information, whereas small filters (1 × 1) can extract local information.

#### 3.2.1. Convolutional Layers

Convolutional layers are one of the main building blocks of CNNs, where their main purpose is to extract features, which they accomplish by convolving a set of features to align with the input feature map. The convolution operator is shift invariant, whereby the spatial relationships between pixels are retained in the output feature map. However, the convolution operator is not invariant to rotation.

#### 3.2.2. Activation Function

After each of the convolutional layers, it is common practice to apply a nonlinear activation function. The activation function performs a fixed mathematical operation on each entry of its inputs, which serves to introduce non-linearities into the network. There are several alternative activation functions, among them the rectified linear unit (ReLU), the use of which has become popular in CNNs, due to its fast computation time, and because it solves problems with vanishing gradients from activation functions such as the hyperbolic tangent or the sigmoid-function. The ReLU is defined as follows:

(1) $$f\left(x\right)=\left\{\begin{array}{cc} x, & \mathrm{if}\;x>0\\ 0, & \mathrm{otherwise} \end{array}\right.$$

#### 3.2.3. Max-Pooling Layer

Max-pooling layers are used for reducing the spatial dimensions of feature maps, which helps the network to learn semantics. The max-pooling layer works by reducing data to a single maximum value within each sliding region of the feature map, thereby reducing the number of parameters (or weights) within the network.

#### 3.2.4. Dropout

Dropout is a simple and powerful regularization technique for neural networks for reducing the risk of overfitting. Dropout is a technique whereby randomly-selected neurons are deactivated during the training phase, thereby forcing the remaining neurons to make up for the dropped out neurons. This helps to share the responsibility across the neurons and coerces them into learning more general features, rather than remembering the specific input.

#### 3.2.5. Average Pooling Layer

In this Inception-v3 architecture, after several convolutional and max-pooling layers, an average pooling layer is used to reduce the computation complexity. An average pooling layer performs a down-sampling by dividing the input into square pooling regions and computing the average values of each region. In this study, the size of the filter for the average pooling layer was 7 × 7.

### 3.3. Fine-Tuning the Network

This network used RGB images as the input, with the input data labeled to enable supervision. The image dataset was divided into two categories: 11,907 images for training and 2516 for testing. During training, a 40% dropout was applied after each of the last two average pooling layers, and in the final layer, the network output was put through a softmax layer, which predicted the weed growth stage. The final softmax layer applies a normalized exponential function to an *N*-dimensional vector, which scales the values of this vector in the range of (0,1) (Equation (2)). This process calculates the confidence in the predicted number of leaves for each image.

(2) $$\sigma \left(z_{j}\right)=\frac{e^{z_{j}}}{\sum_{k=1}^{K}e^{z_{k}}}$$

where j,k∈{1,⋯,K} range over classes, $z_{j}$ refers to the softmax input for class *j* and $\sigma {\left(z\right)}_{j}$ refers to the estimated confidence for class *j*.

The network weights were pre-trained on the ImageNet dataset to take advantage of the general features learned on that dataset already. The training on plant images used a mini-batch of 32 images, where the error of the network was decreased by the adaptive moment estimation (“Adam”) optimizer [30]. Computational efficiency and small memory requirements are some benefits of using the Adam optimizer. Finally, the performance of the network was evaluated by comparison with manually-classified images.

### 3.4. Implementation

All the image processing steps and deep learning methodologies were carried out in Python 3.5 using the Tensorflow 1.4 library.

## 4. Results and Discussion

The following strategies were undertaken in order to analyze leaf counts from 18 different weed species or families. Both the 11,907 images used for training and the 2516 images in the validation set were annotated by three independent plant weed experts, with cross-validation. The Google Inception-v3 architecture was trained on the 11,907 training images, in order to categorize weeds into nine growth stage classes. In order to get a better analysis of errors and improve the accuracy, the network was trained 20 times on the training dataset. Finally, the predictions of the 20 models were combined in order to boost confidence in the predictions. Using a combination of different models allowed us to utilize various features generated within each training, thereby allowing the growth stage levels in each image to be predicted with more confidence and higher accuracy.

**Figure 5:**
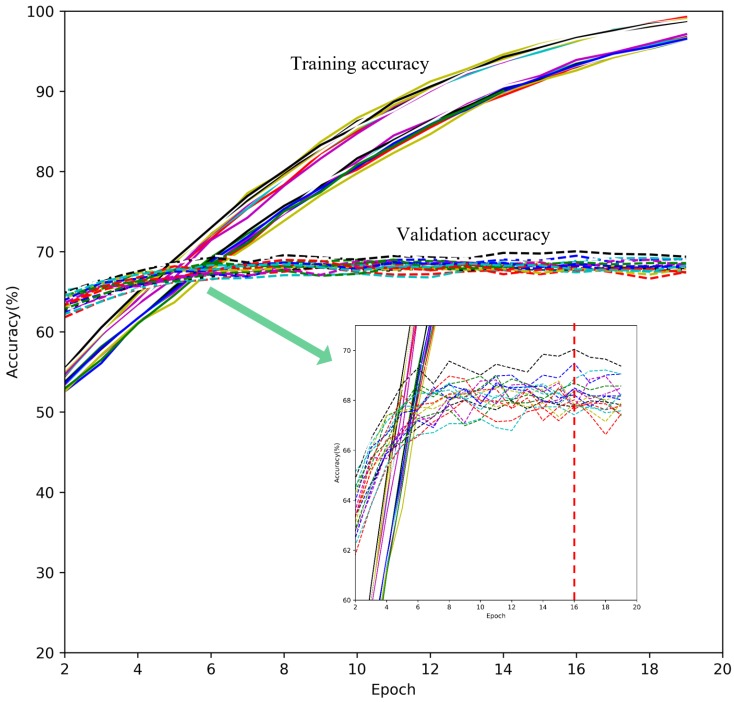
The accuracy progress for the 20 Inception-v3 models.

Figure 5 shows the accuracy for the training and validation sets. The training was stopped after Epoch 16 in order to achieve the highest overall accuracy without overfitting. At this point, the accuracy on the validation set remained constant, and the average accuracy of the 20 models was 70%.

**Figure 6:**
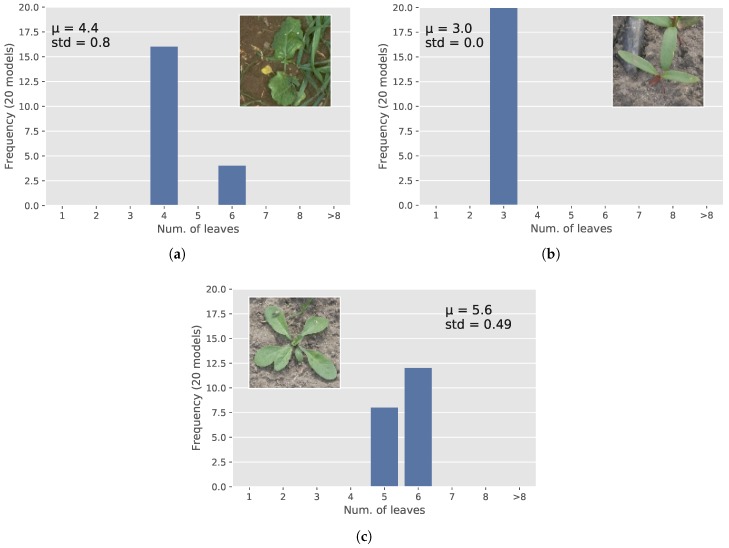
Estimating the number of leaves with different levels of confidence for three images; (**a**) hard case (std = 0.8); (**b**) simple case (std = 0); and (**c**) normal case (std = 0.49).

As the 20 models were not identical, they could predict different growth-stages for the same plants as illustrated in Figure 6. Growth stage predictions of the same plant from different models were more diverse when more plants were present in an image, resulting in a higher standard deviation for those images (Figure 6). In order to enhance the overall accuracy, the softmax-outputs of all 20 models were aggregated, and the index of the maximum value was used as the prediction of the class of a sample.

**Figure 7:**
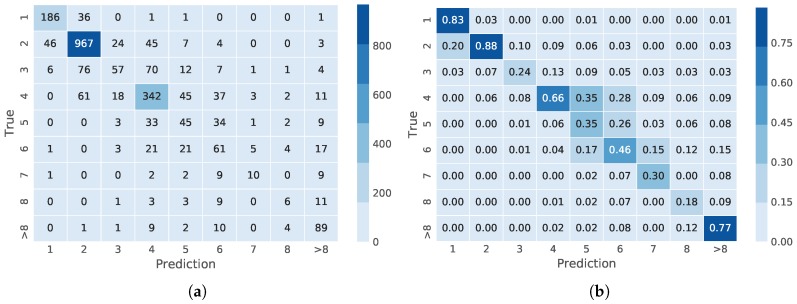
Distribution of predicted growth stages of weeds. (**a**) confusion matrix; (**b**) normalized confusion matrix.

The distribution of predictions is shown in the confusion matrices in Figure 7. These confusion matrices show that for plants with one or two leaves, the accuracy of the 20 combined models was 83% and 88%, respectively. Furthermore, for most of the misclassified images, the errors were either +1 or −1 of the correct value; for example, in the class consisting of just one leaf, 186 images are classified correctly, while 36 images are misclassified as belonging to Class 2, and only one image is misclassified as Class >8. The accuracies for images with 3, 5 or 7 leaves were 24%, 35% and 30%, respectively, but it is worth noting that most of the incorrectly-classified images in these classes are also distributed near the diagonal of the confusion matrix, which indicates that predictions are often close to the correct class. Finally, the accuracies for the plants containing four and >8 leaves were 66% and 77%, respectively. Consequently, classes that have a higher number of samples for training also had a higher accuracy in the validation phase.

**Figure 8:**
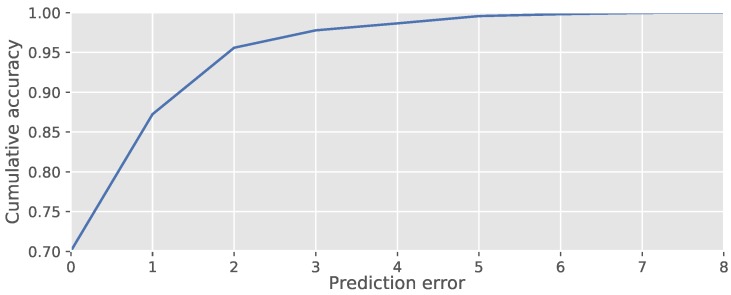
Fraction of plants where the estimated growth stage has a deviation of up to *x* in the counted number of leaves.

Numbers close to the diagonal in the confusion matrix indicate that mistakes made by the leaf predictor are often close to the true number of leaves. Figure 8 shows that 87% of plants have an error of up to one leaf and that 96% of plants have an error of up to two leaves.

Table 1 sums up the achieved results. CountDiff is the average bias of the predictions, which is 0.07 leaves. This indicates that the model has a small tendency of overestimating the number of leaves. The Abs. CountDiff shows that on average, the model is off by 0.51 leaves, and for 70% of the samples, it is off by zero.

Therefore, according to the results obtained, it can be concluded that the developed model can be implemented with 70% accuracy on variable field machines, including machine weed control; because in these systems, the amount of toxic material applied to the weed in the classes of close ranges (e.g., 2:3 or 7:8) does not differ greatly [31].

**Figure 9:**
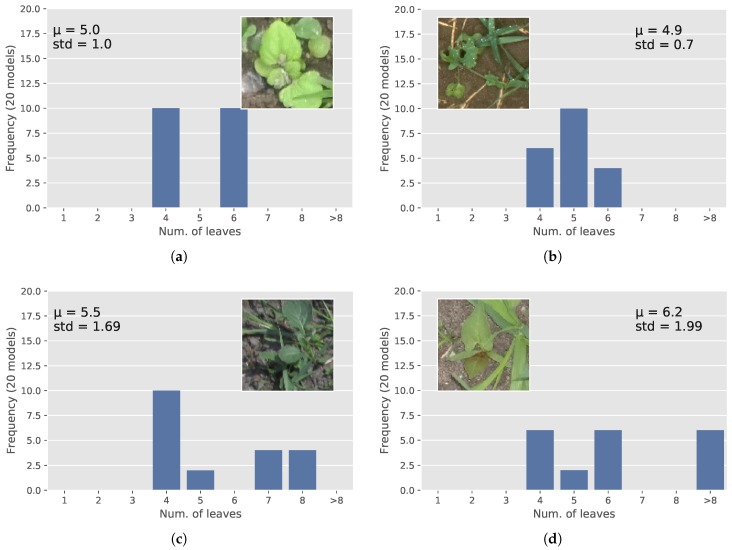
Some hard cases that the models could not classify correctly. Correct labels are: (**a**) four; (**b**) four; (**c**) four; (**d**) four.

Figure 9 shows image samples that were hard to classify with the trained models, which is shown by the distribution of the predictions. However, for these samples, we do not know the correct labels, because there were alternate annotations for the number of leaves from the three different experts that annotated the images: if two experts classified an image with the same number of leaves, we would select this label as a true label; whereas for some cases, all three experts had completely different opinions on counting leaves, and we ignored these images during training and evaluation. Common for such samples is that they have partly- or fully-hidden leaves. We, however, believe that the divergence in predictions of the 20 models can be reduced if the number of hard samples is increased and if even more experts were to annotate samples where there is disagreement between experts.

In order to estimate the confidence interval for each of the CNN predictions of weed species, bootstrapping was used. The probability of an image, $x_{i}$, being classified correctly is given by $P(x_{i})=p$, where *p* is one for a correct classification and *p* is zero for an incorrect classification. The confidence interval was measured by Equation (3):

(3) $$p\left(k\right)=\left(\begin{array}{c} n\\ k \end{array}\right)=p^{k}{\left(1-p\right)}^{n-k}$$

where *k* is the number of correctly-classified images in each weed category and *n* is the number of samples; i.e., k=0,1,2,⋯,n. Confidence in the estimated accuracy was calculated using Wilson’s confidence intervals across 10,000 iterations. The mean accuracy along with confidence intervals for all weed species is presented in Table 2. The best network performance was obtained for Weed Species #1, 7, 10, 12 and 14 (represented at 9, 7, 8, 7 and 9 different growth stages, respectively), probably due to their large number of training samples and their shared physical properties (Table 2). However, for Species #5, 6 and 16, the mean accuracies were 46%, 46% and 56%, respectively; the reason for this lower accuracy is probably due to the lower number of training images and the physical complexity of these species. Furthermore, Table 2 shows a correlation between high variance in achieved accuracies for species with few images in the validation set. The accuracy for Species #6, 11 and 18 is expected to be improved when more training data are available, so they can contribute more to the overall training loss of the network. Moreover, dicotyledons were found to be classified correctly more often than grasses, probably due to their more varied growth habit.

### 4.1. Comparison with Alternative State-Of-The-Art Leaf-Counting Methods

Other researchers who have worked on leaf-counting methods include Ren and Zemel [18] and Romera-Paredes and Torr [19]. While their methods achieved good results on segmented images from the CVPPPP2017 dataset [4], they are difficult to adapt to new environments, as they require fully-segmented leaf images. In contrast the methods proposed by Midtiby et al. [10], Aich and Stavness [21] and Giuffrida et al. [9] only require an image and label as inputs. Likewise, the method outlined in this paper takes as inputs only an image and a label. However, the method by Midtiby et al. [10] works only on binary images without occlusion and cannot, therefore, be applied directly to our images.

The convolutional encoder-decoder network, proposed by Aich and Stavness [21], requires only raw RGB images and the number of leaves, similar to our method. In order to do a direct comparison, our model was retrained on the CVPPP2017 dataset [4], which contains sample images with 31 different numbers of leaves.

Table 1 shows the results of our method applied to the CVPPP2017 dataset. All images from the five directories in the CVPPP2017 dataset (A1–A5) were merged, and a random assortment of 168 images was subtracted as a test set. The average difference in counted leaves (CountDiff) was 0.52, meaning that our method tended to overestimate the number of leaves. However, Aich and Stavness [21] overestimated the number of leaves by 0.73. We obtained an absolute difference in counted leaves (Abs. CountDiff) of 1.31 (Table 1). Finally, the overall accuracy using our method was 41% on the CVPPP2017 data, which was a huge improvement from the 24% obtained by Aich and Stavness [21]. However, It should be noted that the test-set was sampled randomly in both instances and therefore differs between the two studies.

## 5. Conclusions

This study presents a convolutional neural network-based method for estimating the growth stage in terms of number of leaves of various weed species. Images from various camera models were collected in fields with different soil types and light conditions. Because the images were collected under field conditions, plants often overlapped each other, which this network was typically able to overcome. The images spanned 18 common Danish weed species or families, including both monocots and dicots. The average accuracy for these species was 70%, whereas the network achieved an accuracy of 87% if we accept within ±1 of the true growth stage.

When evaluating the network on a per-species level, the highest accuracies were achieved for *Polygonum* (represented at nine different growth stages) and common field speedwell (represented at eight different growth stages), where the accuracies were 78% and 74%, respectively. Whereas for blackgrass and the fine grasses (each represented at nine different growth stages), an accuracy of only 46% was achieved.

Because of the ability to estimate growth stages of weeds, this method is deemed suitable for use in combination with weed detection and classification methods as a support tool when conducting field-based weed control.

## Figures and Tables

**Table 1:** Overall results of our method across all weed species in our dataset and against Aich and Stavness [21] on the CVPPP2017 dataset.

**Dataset**	Our dataset	CVPPP2017 dataset	
**Method**	Ours	Ours	Aich and Stavness [21]
**CountDiff**	0.07	0.52	0.73
**Abs. CountDiff**	0.51	1.31	1.62
**Accuracy**	0.70	0.41	0.24

**Table 2:** Evaluating the accuracies of different weed species using Wilson’s confidence approach with 10,000 iterations in the validation phase.

#	Weed Species	Number of Images	Different Classes	Accuracy	95% CI
1	Common field speedwell	201	9	0.74	0.68–0.80
2	Field pansy	159	8	0.59	0.52–0.67
3	Common chickweed	122	6	0.62	0.52–0.71
4	Fat-hen	102	8	0.62	0.52–0.71
5	Fine grasses (annual meadow-grass, loose silky-bent)	169	9	0.46	0.38–0.53
6	Blackgrass	82	9	0.46	0.35–0.57
7	Hemp-nettle	95	7	0.75	0.66–0.83
8	Shepherd’s purse	76	7	0.64	0.54–0.75
9	Common fumitory	84	7	0.64	0.55–0.74
10	Scentless mayweed	71	8	0.72	0.59–0.82
11	Cereal	66	5	0.54	0.42–0.68
12	Brassicaceae	507	7	0.83	0.80–0.86
13	Maize	91	4	0.69	0.59–0.78
14	*Polygonum*	250	9	0.78	0.73–0.83
15	Oat, volunteers	185	4	0.90	0.85–0.94
16	Cranesbill	86	8	0.56	0.45–0.66
17	Dead-nettle	91	6	0.77	0.68–0.86
18	Common poppy	79	6	0.50	0.39–0.61
